# Der Cluster Headache Impact Questionnaire: Kopfschmerzbezogene Beeinträchtigung bei ClusterkopfschmerzpatientInnen messen

**DOI:** 10.1007/s00482-024-00859-1

**Published:** 2025-01-23

**Authors:** Katharina Kamm, Andreas Straube, Ruth Ruscheweyh

**Affiliations:** Neurologische Klinik und Poliklinik, LMU Klinikum, Ludwig-Maximilians-Universität München, Marchioninistraße 15, 81377 München, Deutschland

**Keywords:** Kopfschmerz, Beeinträchtigung, Fragebogen, Lebensqualität, „Patient-reported outcome measure“, Headache, Disability, Questionnaire, Life quality, Patient-reported outcome measure

## Abstract

Der Clusterkopfschmerz ist eine schwere primäre Kopfschmerzerkrankung, die mit einer erheblichen Beeinträchtigung für die Betroffenen einhergehen kann. Der Cluster Headache Impact Questionnaire (CHIQ) ist ein kurzer Fragebogen zur Erfassung der Clusterkopfschmerz-spezifischen Beeinträchtigung. Eine fünfstufige Schweregradeinteilung von „keine oder leichte Beeinträchtigung“ bis „extreme Beeinträchtigung“ wurde auf der Grundlage der Ergebnisse eines englischsprachigen Patientenkollektivs festgelegt. In der vorliegenden Veröffentlichung wurde untersucht, ob sich diese Schweregradeinteilung auf eine deutsche Kohorte übertragen lässt. Dazu wurden Daten von 196 PatientInnen mit chronischem und episodischem Clusterkopfschmerz während einer aktiven Episode untersucht. Die Schweregradeinteilung des CHIQ zeigt auch im deutschen Patientenkollektiv klinisch sinnvolle Ergebnisse; so unterschieden sich die 5 Schweregrade signifikant in Bezug auf Attackenhäufigkeit, Einnahme von Akutmedikation, unspezifische kopfschmerzbezogene Beeinträchtigung (HIT-6) und Lebensqualität (SF-12v2). Interessanterweise gaben 32 der 52 PatientInnen mit episodischem Clusterkopfschmerz außerhalb der aktiven Episode eine Beeinträchtigung oberhalb des untersten Schweregrads an, also eine mindestens mäßiggradige Beeinträchtigung. Zusammenfassend bietet der CHIQ ein kurzes Instrument zur Erfassung der aktuellen Beeinträchtigung bei Clusterkopfschmerz. Die klinische Interpretation wird durch die hier vorgestellte Schweregradeinteilung erleichtert.

**Graphic abstract:**

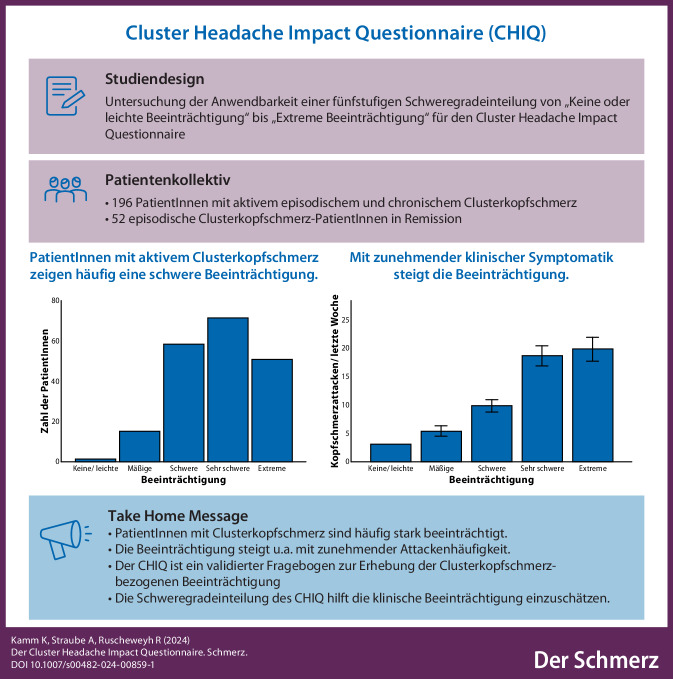

## Abstract (en)

Cluster headache is a severe primary headache disorder, which can be associated with a substantial impairment for sufferers. The Cluster Headache Impact Questionnaire (CHIQ) is a short questionnaire for measuring the cluster headache-specific impairment. A 5-stage severity grading from “no to low impairment” to “’extreme impairment” was established based on the results of an English-speaking patient collective. The present article tested whether the severity classification can be transferred to a German patient group. Data from 196 patients with episodic and chronic cluster headache were examined during an active episode. The severity grading classification of the CHIQ also showed clinically relevant results in the German collective, i.e., the five degrees of severity showed significant differences with respect to attack frequency, intake of acute medication and unspecific headache-related impairment (HIT-6) and quality of life (SF-12v2). Interestingly, 32 out of 52 patients with episodic cluster headache outside the active epísode suffered an impairment above the lowest severity grade, i.e., at least a moderate impairment. In conclusion, the CHIQ provides a short instrument to document the current impairment in cluster headache patients. The severity grading classification presented here facilitates the clinical interpretation.

## Einleitung

Der Clusterkopfschmerz gehört zu den primären Kopfschmerzen [1]. Die Lebenszeitprävalenz liegt zwischen 41,1 und 124/100.000, die jährliche Inzidenz bei 3,0/100.000 [2]. Der Clusterkopfschmerz ist durch extrem starke, oft mehrfach täglich auftretende Kopfschmerzattacken gekennzeichnet, die im angloamerikanischen Sprachraum auch als „Suizidkopfschmerz“ („suicide headache“) bezeichnet werden [3]. Typisch sind streng einseitige Schmerzattacken im Bereich von Auge oder Schläfe, mit einer Dauer zwischen 15 min und 3 h, begleitet von ipsilateralen trigeminoautonomen Symptomen wie Augentränen, Augenrötung, Rhinorrhö oder nasaler Kongestion [1]. Die Unvorhersehbarkeit der Attacken erschwert jegliche Planung. Nächtliche Attacken sind häufig und rauben den PatientInnen den Schlaf [4]. Beim häufigeren episodischen Clusterkopfschmerz wechseln sich Episoden von mehreren Wochen mit regelmäßigen Attacken (jeden 2. Tag bis zu 8 × täglich) mit attackenfreien Phasen von Monaten bis Jahren ab; diese attackenfreien Phasen fehlen beim selteneren chronischen Clusterkopfschmerz. Aus der obigen Beschreibung lässt sich unmittelbar ableiten, dass Clusterkopfschmerzen mit einer großen Beeinträchtigung im Alltag einhergehen können, die bei der Behandlung und Beratung der Betroffenen berücksichtigt werden muss. Für die Erhebung von krankheitsbedingten Auswirkungen auf bspw. die Lebensqualität oder Einschränkungen im Alltag wurden in den letzten Jahren vermehrt „patient-reported outcome measures“ (PROM) eingesetzt, die eine erhebliche Beeinträchtigung von ClusterkopfschmerzpatientInnen zeigten [2, 5]. Zunächst waren dies unspezifische Fragebögen, die krankheitsspezifische Aspekte des Clusterkopfschmerzes nicht berücksichtigen, sodass die tatsächliche Beeinträchtigung der PatientInnen wahrscheinlich unterschätzt wurde [6]. Daher wurden spezifische Bögen für den Clusterkopfschmerz entwickelt: die Clusterkopfschmerzskalen mit 36 Fragen (CHS), die psychosoziale Aspekte erfassen, und ein Clusterkopfschmerz-spezifischer Lebensqualitätsfragebogen mit 28 Fragen (Cluster Headache Quality of Life [CH-QoL]; [7, 8]).

**Abb. 1:**
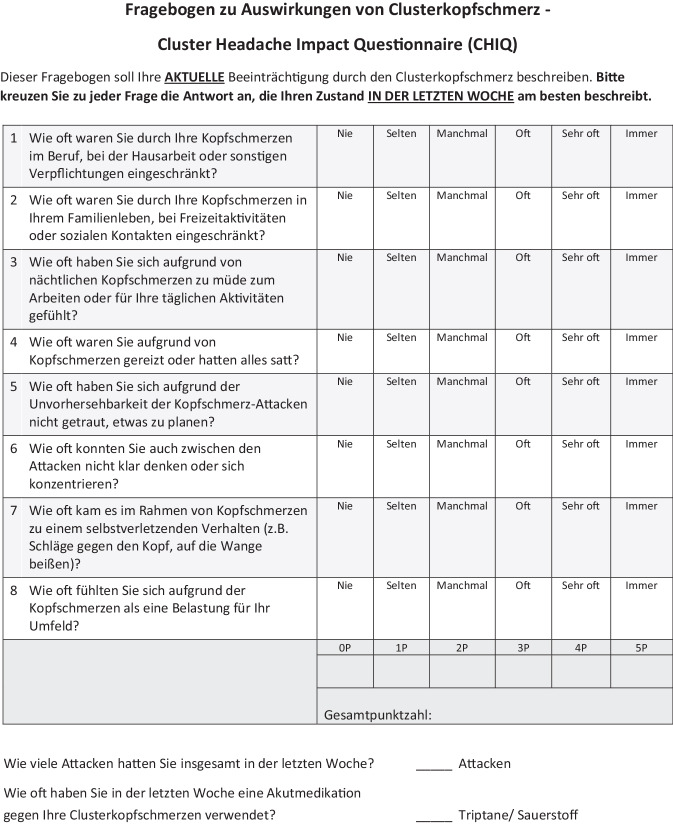
Der deutsche CHIQ-Fragebogen. (Mod. nach [9]). Der Fragebogen ist online abrufbar unter https://cdn.lmu-klinikum.de/d3ddce72fc25221d/dc848893a3a3/CHIQ_Deutsch.pdf

**Abb. 2:**
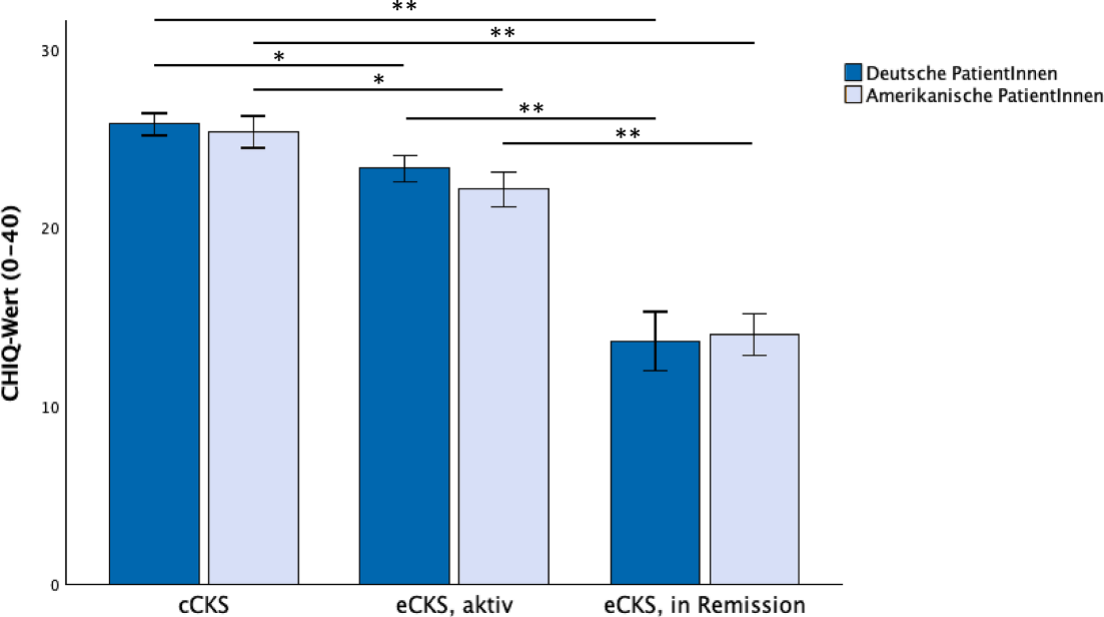
Vergleich der Ergebnisse der CHIQ-Werte in der deutschen (D) und US-amerikanischen (A) Kohorte. Die Abbildung zeigt die CHIQ-Werte für chronische ClusterkopfschmerzpatientInnen (cCKS; D: 25,8 ± 6,5, A: 25,4 ± 7,9), episodische PatientInnen während der Episode (eCKS, aktiv; D: 23,3 ± 6,9, A: 22,2 ± 8,7) und in Remission (eCKS, in Remission; D: 13,6 ± 11,9, A: 14,0 ± 13,1). Es zeigen sich signifikante Unterschiede zwischen den Diagnosegruppen (H (2) = 86,4, *p* < 0,001, Post-hoc-Test mit Bonferroni-Korrektur: *p* < 0,001 für eCKS, in Remission vs. cCKS und vs. eCKS, aktiv; *p* < 0,05 für eCKS, aktiv vs. cCKS), nicht aber zwischen den deutschen und US-amerikanischen PatientInnen in der jeweiligen Gruppe (cCKS: t (185) = 0,406, *p* = 0,685; eCKS, aktiv: t (162) = 0,946, *p* = 0,346; eCKS, in Remission: t (176) = −0,166, *p* = 0,868). ^*^ < 0,05, ^**^ < 0,001

Dies war der Ausgangspunkt für die Entwicklung des dezidiert kurzen *Cluster Headache Impact Questionnaire* (CHIQ), der mit 8 Fragen die Clusterkopfschmerz-spezifische Beeinträchtigung erfasst und insbesondere auf dessen Besonderheiten, wie z. B. das nächtliche Auftreten von Kopfschmerzattacken oder das Vorkommen von selbstverletzendem Verhalten, eingeht (Abb. 1; [9]; Fragebogen abrufbar unter https://cdn.lmu-klinikum.de/d3ddce72fc25221d/dc848893a3a3/CHIQ_Deutsch.pdf). Um die aktuelle Beeinträchtigung zu erheben, bezieht sich der Fragebogen auf die letzte Woche. Der CHIQ wurde ursprünglich auf Deutsch entwickelt und ist mittlerweile auch in englischer und italienischer Sprache validiert [9–11]. In der deutschen und englischen Validierungsstudie zeigten chronische ClusterkopfschmerzpatientInnen die höchste Beeinträchtigung, gefolgt von episodischen ClusterkopfschmerzpatientInnen in der aktiven Episode und, mit deutlichem Abstand, von episodischen PatientInnen in Remission, mit jeweils signifikanten Unterschieden. Interessanterweise waren die Mittelwerte des CHIQ in den 3 Diagnosegruppen bei deutschen und englischsprachigen (US-amerikanischen) PatientInnen sehr ähnlich ([9, 10]; siehe Abb. 2).

In der englischen Validierungsstudie wurde anhand der Ergebnisse aktiver episodischer und chronischer PatientInnen eine fünfstufige Schweregradeinteilung entwickelt, von „keine bis leichte Beeinträchtigung“ bis zu „extreme Beeinträchtigung“ (Tab. 1; [10]). Hierbei wurden zwei Erfordernisse berücksichtigt: Einerseits sollte die Bezeichnung des jeweiligen Schweregrads das Empfinden der PatientInnen widerspiegeln. Dies wurde auf Basis der Ergebnisse einer separaten subjektiven Schweregradeinschätzung der PatientInnen sichergestellt. Andererseits sollte die Schweregradeinteilung eine gute Diskriminierung auch in der oberen Hälfte der CHIQ-Skala ermöglichen, wo die Ergebnisse der meisten PatientInnen mit aktivem Clusterkopfschmerz zu finden sind. Daher wurde der CHIQ-Bereich zwischen 15 und 40 Punkten, in dem sich fast alle Patienten befanden, die sich selbst als „ziemlich stark“ oder „extrem“ „betroffen“ einschätzten, in 3 Gruppen mit etwa gleichen Patientenzahlen eingeteilt, entsprechend den CHIQ-Graden 3 (schwere Beeinträchtigung), 4 (sehr schwere Beeinträchtigung) und 5 (extreme Beeinträchtigung). Details sind bei Kamm et al. beschrieben [10]. Die Validität der so entwickelten Schweregradeinteilung zeigte sich anhand klinischer Charakteristika. So fand sich eine über die Schweregrade ansteigende Attackenhäufigkeit und Häufigkeit der Nutzung von Akutmedikation sowie ansteigende Werte von Ängstlichkeit, Depression und Stress [10]. In der hier vorliegenden Studie überprüften wir, ob die so festgelegten Schweregrade auch in der deutschen Studienpopulation zu einer klinisch sinnvollen Einteilung führen.

**Tab. 1:** Schweregrade des CHIQ. (Nach [10])

CHIQ-Punktzahl	Grad	Bezeichnung
0–4	1	Keine oder leichte Beeinträchtigung
5–14	2	Mäßige Beeinträchtigung
15–23	3	Schwere Beeinträchtigung
24–29	4	Sehr schwere Beeinträchtigung
30–40	5	Extreme Beeinträchtigung

## Methoden

Nach Zustimmung der Ethikkommission der Universität München (20-738) wurde die Studie zwischen Oktober 2020 und Februar 2021 unter Beachtung der Deklaration von Helsinki durchgeführt. Erwachsene PatientInnen mit der Diagnose eines episodischen oder chronischen Clusterkopfschmerzes nach den Kriterien der Internationalen Kopfschmerzklassifikation (International Classification of Headache Disorders [ICHD-3]; [1]) wurden am Oberbayerischen Kopfschmerzzentrum und mit der Unterstützung einer Clusterkopfschmerz-Selbsthilfeorganisation (CSG) rekrutiert. Die PatientInnen konnten entweder online mittels RedCap (Research Electronic Data Capture; [12, 13]) oder mit einem Papierfragebogen teilnehmen. Die Befragung umfasste den CHIQ, einen Anamnesebogen zur Erhebung krankheitsbezogener und demografischer Daten sowie einen generischen Kopfschmerzbeeinträchtigungsbogen (Headache Impact Test [HIT-6]; [14]), die Depression-Angst-Stress-Skalen (DASS [15]) und den Lebensqualitätsfragebogen Short Form 12 (SF-12v2 [16]). Der HIT‑6 ist ein Fragebogen mit 6 Items, um Beeinträchtigung durch Kopfschmerzen zu erheben. Die Fragen beziehen sich auf Einschränkungen im täglichen Leben, ein Rückzugsbedürfnis, eine kopfschmerzbezogene Erschöpfung und Konzentrationsschwierigkeiten [17]. Die DASS umfassen 21 Fragen und bilden 3 Subskalen zu Depression, Angst und Stress [15]. Der SF-12v2 ist ein allgemeiner Lebensqualitätsfragebogen, der 8 Domänen umfasst. Aus diesen werden die beiden Subskalen – die körperliche und psychische Summenskala – berechnet [18]. Alle drei Fragebögen zeigen eine gute Validität und Reliabilität. Die Methoden und Ergebnisse der Validierungsstudie sind detailliert bei Kamm et al. beschrieben [9]. Hier wurde die von Kamm et al. [10] entwickelte Schweregradskala auf diese Daten angewendet.

### Cluster Headache Impact Questionnaire (CHIQ)

Der CHIQ umfasst 8 Fragen (Abb. 1). Zwei Fragen erheben Einschränkungen in Arbeit, Hausarbeit, Familie und Freizeit. Vier Fragen adressieren die mentale und körperliche Beeinträchtigung aufgrund von Konzentrationsschwierigkeiten, Gereiztheit, fehlender Planbarkeit und nächtlichen Attacken. Zwei Fragen erfassen selbstverletzendes Verhalten während der Attacken sowie das Gefühl, eine Last für das soziale Umfeld zu sein. Zusätzlich wird die Attackenhäufigkeit sowie die Einnahme von Akutmedikation (AM) in der letzten Woche erfragt. Die Fragen werden auf einer Skala von „nie“ bis „immer“ beantwortet, denen ein Punktwert von 0 bis 5 Punkten zugeordnet ist. Das bedeutet, dass eine Gesamtpunktzahl zwischen 0 und 40 Punkten erreicht werden kann. Eine höhere Punktzahl geht mit einer größeren Beeinträchtigung einher. Die Schweregrade wurden entsprechend Kamm et al. definiert (Tab. 1). Der CHIQ zeigte sowohl in der deutschen als auch in der englischen Validierungsstudie eine gute interne Konsistenz (D: Cronbachs α = 0,88, A: Cronbachs α = 0,91), Reliabilität (D: ICC = 0,91, A: ICC = 0,93) und Validität [9, 10].

### Statistische Auswertung

Unterschiede zwischen den 5 Schweregraden wurden mittels einer Kruskal-Wallis-ANOVA berechnet, als Post-hoc-Test wurde ein Mann-Whitney-U-Test mit Bonferroni-Korrektur verwendet, dabei wurde nur jede Schweregradgruppe mit der nächsthöheren Schweregradgruppe verglichen. Paarweise Vergleiche mit Schweregradgruppe 1 wurden nicht durchgeführt, da nur ein Patient in diese Gruppe fiel (siehe unten). Die Häufigkeitsverteilung zwischen aktiven ClusterkopfschmerzpatientInnen und ClusterkopfschmerzpatientInnen in Remission wurde mit einem exakten Test nach Fisher berechnet. Für den Vergleich zwischen den Patientengruppen in Schweregradeinteilung 1 wurde ein exakter Binominaltest verwendet. Die statistische Auswertung wurde mit SPSS Statistics 26 (IBM Corp., Armonk, NY, USA) durchgeführt. Ein *p*-Wert < 0,05 (zweiseitig) wurde als signifikant gewertet.

## Ergebnisse

**Abb. 3:**
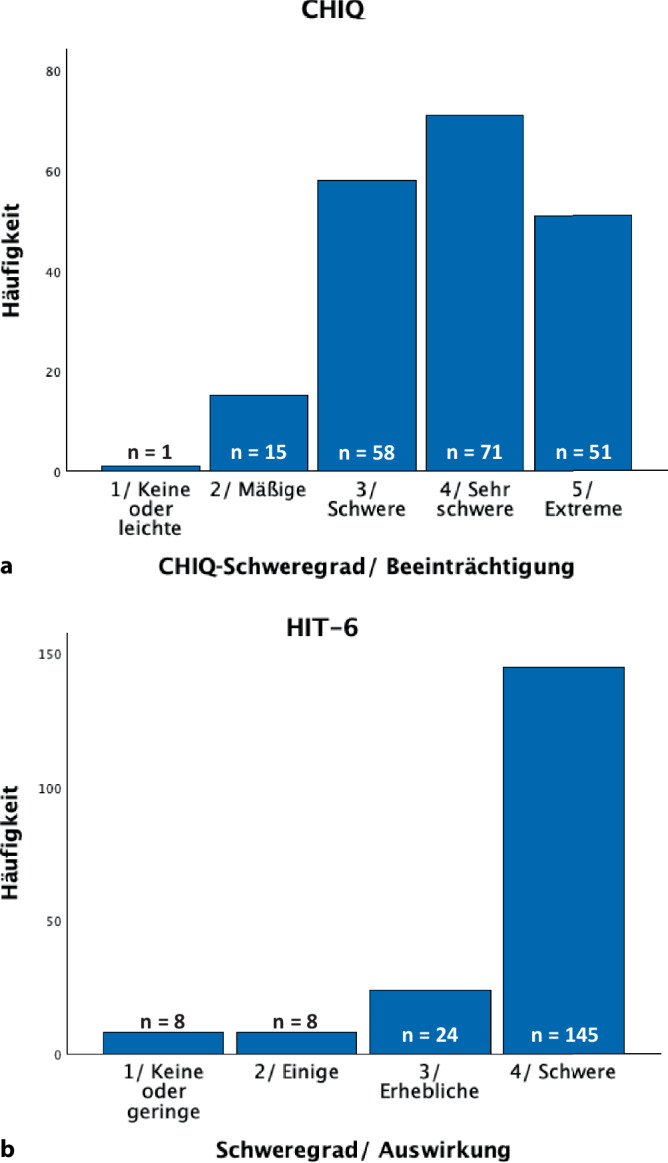
Verteilung über die unterschiedlichen Schweregrade in CHIQ (**a**) und HIT-6 (**b**). **a** Aktive episodische und chronische ClusterkopfschmerzpatientInnen zeigen eine gute Verteilung über die einzelnen Schweregrade mit guter Differenzierung bei starker Beeinträchtigung. **b** Die überwiegende Mehrheit der PatientInnen ist schwer betroffen, in HIT‑6 findet kaum eine Differenzierung zwischen den stärker betroffenen PatientInnen statt

196 chronische und episodische ClusterkopfschmerzpatientInnen in der aktiven Episode (episodisch *n* = 85, männlich *n* = 128; 47,2 ± 11,6 Jahre) wurden in die Auswertung eingeschlossen (Tab. 2). Der mittlere CHIQ-Wert lag bei 24,7 ± 6,8. Die PatientInnen verteilten sich wie folgt auf die 5 Schweregrade: „keine oder leichte Beeinträchtigung“: 1 Patient (0,5 %); „mäßige Beeinträchtigung“: 15 PatientInnen (7,7 %), „schwere Beeinträchtigung“: 58 (29,6 %), „sehr schwere Beeinträchtigung“: 71 (36,2 %) und „extreme Beeinträchtigung“: 51 PatientInnen (26,2 %; Abb. 3a). Charakteristika der Gruppen sind in Tab. 3 dargestellt.

**Tab. 2:** Übersicht über die deutschen PatientInnen

	cCKS-PatientInnen	Aktive eCKS-PatientInnen	eCKS-PatientInnen, in Remission
*n* (männlich)	111 (58)	85 (70)	52 (39)
Alter (Jahre)	48,1 ± 11,6	46,1 ± 11,7	48,7 ± 10,9
Erkrankungsdauer (Jahre)	15,5 ± 9,5	17,2 ± 11,7	21,2 ± 10,3
Attackenhäufigkeit^*^	16,5 ± 13,7	13,4 ± 13,7	0
Nächtl. Attacken, *n* (%)	102 (91,9 %)	79 (92,9 %)	43 (82,7 %)^$^
Kopfschmerzintensität (0–10)	7,6 ± 2,1	7,8 ± 1,7	8,0 ± 2,1^$^
Einnahme von Akutmedikation, *n* (%)	105 (94,6 %)	77 (90,6 %)	–
Häufigkeit der Akutmedikation^+^	14,3 ± 13,7	12,5 ± 14,8	–
Einnahme prophylaktischer Medikation, *n* (%)	76 (68,5 %)	44 (51,8 %)	–

*cCKS* chronischer Clusterkopfschmerz, *eCKS* episodischer Clusterkopfschmerz

*Attacken in der letzten Woche

^+^Einnahmehäufigkeit der Akutmedikation in der letzten Woche

^$^Die Angaben beziehen sich auf die letzte Episode (vgl. [9])

**Tab. 3:** Schweregradeinteilung des CHIQ. Gruppenvergleiche wurden mit einer Kruskal-Wallis-ANOVA und Post-hoc-Tests wurden mittels Mann-Whitney-U-Test mit Bonferroni-Korrektur durchgeführt

Einteilung	Grad 1	Grad 2	Grad 3	Grad 4	Grad 5	Statistik
	Keine oder leichte Beeinträchtigung	Mäßige Beeinträchtigung	Schwere Beeinträchtigung	Sehr schwere Beeinträchtigung	Extreme Beeinträchtigung	
CHIQ-Punktzahl	0–4	5–14	15–23	24–29	30–40	–
*N*	1	15	58	71	51	–
⌀ CHIQ	2,0	11,1 ± 2,6	19,8 ± 2,8	26,5 ± 1,7	32,4 ± 2,2	–
Kopfschmerzattacken/letzte Woche	3,0	5,3 ± 3,6	9,7 ± 8,7	18,6 ± 14,9**	19,7 ± 15,2	H (4) = 34,9, *p* < 0,001
Einnahme von Akutmedikation/letzte Woche	3,0	4,8 ± 4,1	8,0 ± 7,9	16,1 ± 15,2**	18,9 ± 16,9	H (4) = 28,2, *p* < 0,001
HIT‑6	44,0	54,9 ± 6,4	60,9 ± 5,9*	63,5 ± 4,7	68,0 ± 4,8**	H (4) = 59,2, *p* < 0,001
DASS Depression	1,0	4,0 ± 3,0	6,6 ± 4,2	9,3 ± 4,6	13,6 ± 4,5**	H (4) = 59,3, *p* < 0,001
DASS Angst	1,0	3,2 ± 2,4	4,4 ± 3,5	6,1 ± 4,5	9,5 ± 4,6**	H (4) = 39,2, *p* < 0,001
DASS Stress	3,0	4,2 ± 2,9	8,2 ± 4,5*	10,3 ± 4,6	14,0 ± 4,4**	H (4) = 52,3, *p* < 0,001
SF-12v2 Körperliche Skala (PCS)	58,4	48,0 ± 7,4	45,9 ± 7,9	39,7 ± 8,4**	37,6 ± 6,9	H (4) = 36,5, *p* < 0,001
SF-12v2 Psychische Skala (MCS)	54,6	44,9 ± 8,3	41,2 ± 10,3	36,3 ± 9,7*	30,8 ± 7,0*	H (4) = 38,7, *p* < 0,001

*p < 0,05

**p < 0,001 für den Vergleich mit dem jeweils nächst niedrigeren Schweregrad

**Abb. 4:**
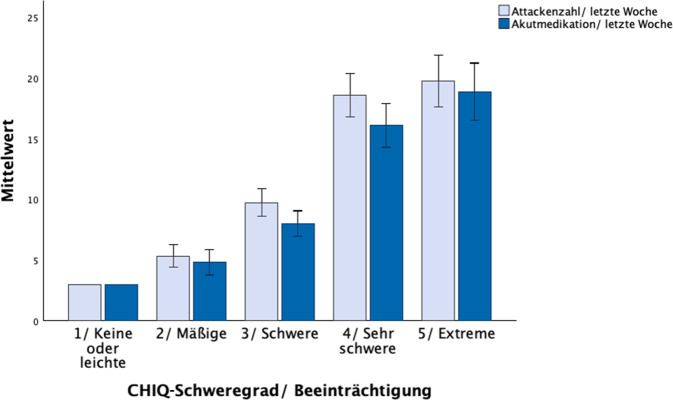
Ein höherer CHIQ-Schweregrad geht mit einer höheren Attackenzahl und häufigeren Einnahme von Akutmedikation einher. Die Unterschiede zwischen den Graden sind signifikant (Attackenhäufigkeit: H (4) = 34,9, *p* < 0,001; Einnahmehäufigkeit von Akutmedikation: H (4) = 28,2, *p* < 0,001)

Die Schweregradgruppen unterschieden sich signifikant in der Attackenhäufigkeit (H (4) = 34,9, *p* < 0,001) und Einnahmehäufigkeit von Akutmedikation (H (4) = 28,2, *p* < 0,001), wobei sich ein monotoner Anstieg über die Schweregrade zeigt (Abb. 4). Die Post-hoc-Analyse zeigte einen signifikanten Unterschied zwischen der Gruppe „schwere Beeinträchtigung“ und „sehr schwere Beeinträchtigung“ (Attackenhäufigkeit: *p* < 0,001; Einnahmehäufigkeit von Akutmedikation: *p* < 0,001, Tab. 3).

**Abb. 5:**
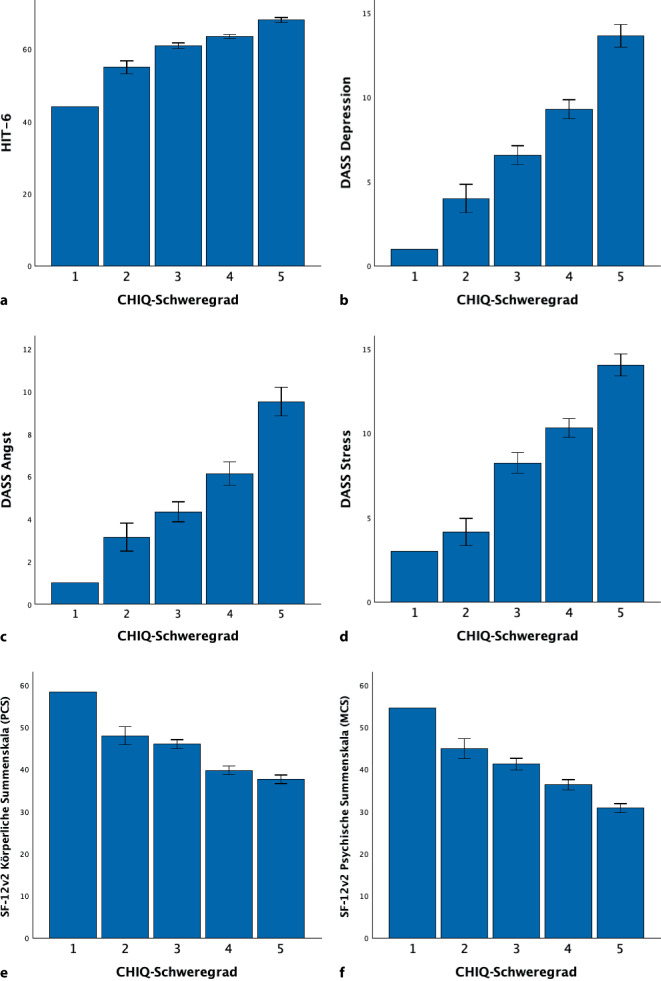
Die Beeinträchtigungsgrade im CHIQ zeigten signifikante Unterschiede hinsichtlich der kopfschmerzbezogenen Beeinträchtigung (HIT‑6; **a**), in Bezug auf Depression (DASS‑D; **b**), Angst (DASS‑C; **c**), Stress (DASS‑D; **d**) sowie Lebensqualität (SF-12v2; **e** und **f**). **a** PatientInnen mit einem höheren CHIQ-Grad zeigten eine größere Beeinträchtigung in HIT‑6 (HIT‑6, H (4) = 59,2, *p* < 0,001). **b**, **c**, **d** Signifikante Unterschiede zeigten sich auch in den DASS. Ein höherer CHIQ-Grad ging mit einer höheren Depressivität (H (4) = 59,3, *p* < 0,001), mehr Angst (H (4) = 39,2, *p* < 0,001) und Stress (H (4) = 52,3, *p* < 0,001) einher. **e**, **f** Ein höherer CHIQ-Grad ging mit einer signifikant niedrigeren Lebensqualität (SF-12v2 PCS, H (4) = 36,5, *p* < 0,001; SF-12v2 MCS, H (4) = 38,7, *p* < 0,001) einher

Ebenso zeigten sich signifikante, monotone Anstiege in der unspezifischen kopfschmerzbezogenen Beeinträchtigung (HIT‑6, H (4) = 59,2, *p* < 0,001) sowie für Symptome von Depression, Angst und Stress (DASS Depression, H (4) = 59,3, *p* < 0,001; DASS Angst, H (4) = 39,2, *p* < 0,001, DASS Stress, H (4) = 52,3, *p* < 0,001) über die Schweregrade (Abb. 5). Signifikante Unterschiede zeigten sich auch in Bezug auf die Lebensqualität. Die beiden Summenwerte Körperliche (PCS) und Psychische Summenskala (MCS) von SF-12v2 zeigten mit höherem CHIQ-Schweregrad signifikant niedrigere Summenwerte (SF-12v2 PCS, H (4) = 36,5, *p* < 0,001; SF-12v2 MCS, H (4) = 38,7, *p* < 0,001, Abb. 5). In den Post-hoc-Tests zeigten sich signifikante Unterschiede zwischen den Gruppen „sehr schwere Beeinträchtigung“ und „extreme Beeinträchtigung“ für HIT‑6 (*p* < 0,001) sowie für DASS Depression (*p* < 0,001), DASS Angst (*p* < 0,001), DASS Stress (*p* < 0,001) und die Psychische Summenskala von SF-12v2 (*p* < 0,012, Tab. 3). Weiterhin zeigten sich signifikante Unterschiede zwischen den Gruppen „mäßige Beeinträchtigung“ und „schwere Beeinträchtigung“ für HIT‑6 (*p* = 0,027) und DASS Stress (*p* = 0,021) und zwischen den Gruppen „schwere Beeinträchtigung“ und „sehr schwere Beeinträchtigung“ für die Psychische und Körperliche Summenskala von SF-12v2 (MCS: *p* = 0,021; PCS: *p* < 0,001).

Die deutsche Kohorte enthielt auch 52 PatientInnen mit einem episodischen Clusterkopfschmerz in Remission (während einer attackenfreien Phase). Davon fielen 20 PatientInnen (38,5 %) in die Gruppe „keine oder leichte Beeinträchtigung“, 4 PatientInnen (7,7 %) erreichten eine „mäßige Beeinträchtigung“, gefolgt von 15, 8 und 5 PatientInnen (28,8 %, 15,4 % und 9,6 %) in den Gruppen „schwere Beeinträchtigung“, „sehr schwere Beeinträchtigung“ und „extreme Beeinträchtigung“. Diese Verteilung ist signifikant unterschiedlich zu der Verteilung der PatientInnen mit aktivem Clusterkopfschmerz über die Schweregrade (*p* = 0,001), insbesondere bezüglich der Anzahl der PatientInnen in Grad 1 (38,5 % vs. 0,5 %, *p* = 0,001, *n* = 21).

## Diskussion

Das Ergebnis der Studie ist, dass sich die in der US-amerikanischen Stichprobe entwickelte Schweregradeinteilung des CHIQ auf die deutsche Stichprobe übertragen lässt. Die Verteilung über die Gruppen war ähnlich der US-amerikanischen Stichprobe mit einem Hauptteil der PatientInnen (91,8 %) in den oberen drei Schweregraden. Die Verteilung mit 58, 71 und 51 PatientInnen über die drei oberen Schweregrade zeigt, dass eine ungefähre Gleichverteilung ohne ausgeprägte Ceiling-Effekte gelungen ist (Abb. 3a). Dagegen diskriminiert der HIT-6-Test nur unzureichend zwischen ClusterkopfschmerzpatientInnen (Abb. 3b). Ebenso findet man z. B. in schwer betroffenen Kollektiven von MigränepatientInnen im Migraine Disability Assessment (MIDAS; [19]) oft > 60 % der PatientInnen in Grad 4 (von 4), sodass eine Diskriminierung im oberen Bereich nicht möglich ist (z. B. [20]). Dies konnte hier vermieden werden.

Die Validität der Schweregradeinteilung zeigt sich außerdem darin, dass für alle klinischen Parameter und die Ergebnisse der Fragebögen zu unspezifischer kopfschmerzbezogener Beeinträchtigung (HIT-6), Lebensqualität (SF-12v2) sowie Depression, Angst und Stress (DASS) ein monotoner Verlauf mit über die Schweregrade zunehmender Symptomatik oder Ausprägung bzw. schlechterer Lebensqualität festzustellen war.

Die hier beschriebene Schweregradeinteilung kann Behandler dabei unterstützen, die Schwere der Beeinträchtigung der PatientInnen besser einzuschätzen und entsprechende Konsequenzen zu ziehen – z. B. bezüglich der Dringlichkeit einer schnell und stark wirksamen medikamentösen Behandlung (gegenüber einer nebenwirkungsärmeren, aber weniger schnell wirksamen Behandlung), aber auch bezüglich der Notwendigkeit einer unterstützenden Psychotherapie. Andererseits bringt eine Schweregradeinteilung immer einen Informationsverlust gegenüber den Rohwerten mit sich, sodass idealerweise v. a. im Verlauf beides, Schweregrad und CHIQ-Summenscore, betrachtet werden sollte. Die Veränderungssensitivität des CHIQ ist aktuell noch Gegenstand von Untersuchungen.

Lebensqualität, psychosoziale Faktoren und kopfschmerzspezifische Beeinträchtigung wurden bei ClusterkopfschmerzpatientInnen bis vor Kurzem wenig und oftmals mit unspezifischen, für diese Gruppe nicht validierten Instrumenten untersucht [5, 21]. Das episodenhafte Auftreten mit häufig wechselnder Attackenfrequenz sowie nächtlichen Attacken erschwert die Erfassung. In früheren Studien wurden häufig kleine und schwer betroffene Patientengruppen untersucht, sodass die Ergebnisse nur wenig verallgemeinert werden konnten [21]. Darüber hinaus muss die Verwendung allgemeiner Lebensqualitätsfragebögen oder generischer Fragebögen zur Erhebung der Beeinträchtigung durch Kopfschmerz kritisch gesehen werden, da diese die spezifischen Charakteristika der Erkrankung nicht ausreichend berücksichtigen und entsprechend die tatsächliche Beeinträchtigung wahrscheinlich unterschätzen. Dennoch weisen auch frühere Studien auf eine geringere Lebensqualität und höhere Beeinträchtigung von ClusterkopfschmerzpatientInnen im Vergleich zu gesunden ProbandInnen oder PatientInnen mit einer anderen Kopfschmerzerkrankung hin [5, 21].

Durch die Entwicklung von zwei längeren Fragebögen für die Erhebung der Clusterkopfschmerz-spezifischen Lebensqualität (CH QoL) sowie der psychosozialen Faktoren bei Clusterkopfschmerz (CHS) konnten die o. g. Ergebnisse in größeren und heterogeneren CKS-Patientengruppen bestätigt werden [7, 8]. Die CHS wurden auf Deutsch entwickelt und validiert, auch die deutsche Übersetzung des CH QoL ist inzwischen validiert und die Änderungssensitivität des CH QoL wurde anhand einer kleinen Stichprobe (*n* = 10) untersucht [22, 23]. Auch diese Studien zeigen, dass chronische CKS-PatientInnen eine niedrigere Lebensqualität und eine höhere Beeinträchtigung im Vergleich zu episodischen ClusterkopfschmerzpatientInnen und gesunden Kontrollen aufweisen.

Der CHIQ ergänzt diese ausführlichen Instrumente als dezidiert kurzer Clusterkopfschmerz-spezifischer Beeinträchtigungsfragebogen. Er ist bereits in deutscher, englischer und italienischer Sprache validiert und korreliert gut mit klinischen Parametern (z. B. Attackenhäufigkeit), Lebensqualität und psychologischen Kofaktoren. Es zeigte sich, dass chronische ClusterkopfschmerzpatientInnen die höchsten CHIQ-Werte und damit die höchste Beeinträchtigung aufwiesen, gefolgt von aktiven, episodischen ClusterkopfschmerzpatientInnen und episodischen ClusterkopfschmerzpatientInnen in Remission. Interessanterweise gaben auch episodische ClusterkopfschmerzpatientInnen außerhalb der Episode eine Beeinträchtigung an [9, 10], die auch bereits in einer früheren Studie beschrieben wurde [6]. Dies sieht man auch in der hier beschriebenen Schweregradeinteilung daran, dass episodische PatientInnen in Remission nicht alle in Schweregrad 1 fallen, sondern durchaus auch in höheren Schweregraden zu finden sind. Dies könnte bspw. durch die Angst vor erneuten Attacken, wie sie auch in den CHS beschrieben wird, oder durch anhaltende Auswirkungen der Erkrankung auf die Lebensqualität bedingt sein [8]. In einer kürzlich veröffentlichten Studie zeigten u. a. die Kopfschmerzhäufigkeit und die Verlaufsform der Erkrankung einen Einfluss auf die Lebensqualität außerhalb der Attacken. Die Autoren schlussfolgerten, dass ein besseres Verständnis der interiktalen Belastungen unerlässlich für eine umfassende Behandlung der PatientInnen ist [24]. Um dies genauer zu verstehen, sind weitere Studien notwendig.

Aufgrund seiner günstigen Eigenschaften hat der CHIQ bereits Eingang in das Kopfschmerzregister der Deutschen Migräne- und Kopfschmerzgesellschaft (DMKG) sowie in den DMKG-Kopfschmerzfragebogen (www.dmkg.de) gefunden [25].

## Limitationen

Die vorliegende Studie hat einige Limitationen, die in den vorherigen Veröffentlichungen ausführlich diskutiert sind [9, 10]. Bei PatientInnen der Selbsthilfegruppe basierte die Diagnose zunächst auf dem Selbstbericht, wurde jedoch durch Abfrage der ICHD-3-Kriterien überprüft. Die Einbeziehung von PatientInnen einer Selbsthilfegruppe erlaubte, eine breitere Stichprobe zu untersuchen (im Vergleich zur Beschränkung auf PatientInnen eines tertiären Zentrums), auch wenn wahrscheinlich weiterhin ein Bias hin zu schwerer betroffenen PatientInnen besteht. Die Stichprobe ist mit 196 aktiven PatientInnen für eine Clusterkopfschmerzstudie groß, jedoch fanden sich bei den aktiven PatientInnen in den unteren Schweregradgruppen kleine Zahlen, sodass die paarweisen Vergleiche mit Schweregrad 1 oder 2 nicht möglich waren bzw. oft nicht signifikant wurden.

## Ausblick

Zusammenfassend ist der CHIQ ein kurzer, für den klinischen Alltag und für Studien geeigneter Fragebogen zur Erhebung der Clusterkopfschmerz-spezifischen Beeinträchtigung. Die hier vorgestellte Schweregradeinteilung hilft bei der Einstufung der Beeinträchtigung.

## Fazit für die Praxis

- Der CHIQ ist ein kurzer Fragebogen, um die Beeinträchtigung bei ClusterkopfschmerzpatientInnen zu erheben.
- Aktive ClusterkopfschmerzpatientInnen zeigen eine hohe Beeinträchtigung.
- Mit höherer Beeinträchtigung nehmen Depressivität, Angst und Stress zu und die Lebensqualität ab.

## Data Availability

Die Daten, die die Ergebnisse dieser Publikation stützen, sind auf begründete Anfrage beim korrespondierenden Autor erhältlich.
